# Zoledronic Acid for Periprosthetic Bone Mineral Density Changes in Patients With Osteoporosis After Hip Arthroplasty—An Updated Meta-Analysis of Six Randomized Controlled Trials

**DOI:** 10.3389/fmed.2021.801282

**Published:** 2021-12-23

**Authors:** Yuan Liu, Jia-Wen Xu, Ming-Yang Li, Li-Min Wu, Yi Zeng, Bin Shen

**Affiliations:** Department of Orthopedics, National Clinical Research Center for Geriatrics, Orthopedics Research Institute, West China Hospital, Sichuan University, Chengdu, China

**Keywords:** zoledronic acid, total hip arthroplasty, osteoporosis, systematic review, meta-analysis, randomized controlled trial

## Abstract

**Introduction:** Periprosthetic bone mineral density (BMD) loss following total hip arthroplasty (THA) may threaten the survival of the implant, especially in patients with osteoporosis. Zoledronic acid (ZA) is the representative of the third generation of bisphosphonates, which were effective in reducing bone loss in conditions associated with accelerated bone turnover. The aim of this study was to evaluate the efficacy and safety of ZA in patients with osteoporosis after THA.

**Methods:** Randomized controlled trials (RCTs) associated with ZA and THA were searched from the MEDLINE, PubMed, EMBASE, Wanfang database, and the Web of Science (August 2021). Other methods, such as hand search and email request were also tried. The methodological quality was assessed by the Risk of Bias (RoB) 2.0. Relevant data were abstracted from the included RCTs and authors were contacted when necessary.

**Results:** In this study, six RCTs involving a total of 307 patients were finally included and analyzed. The pooled data demonstrated that significantly less periprosthetic BMD loss in Gruen zone seven had occurred in the ZA-treated patients than in the control patients at 3 months (mean difference [MD] = 4.03%; 95% *CI*: 0.29–7.76%; *P* = 0.03), 6 months (MD = 7.04%; 95% *CI*: 2.12–11.96%; *P* = 0.005), and 12 months (MD = 7.12%; 95% *CI*: 0.33–13.92%; *P* = 0.04). The Harris Hip Score (HHS) was also significantly increased in ZA group at 6 and 12 months after operation (*P* = 0.03 and *P* = 0.02, respectively). Influenza-like symptom was found related to the usage of ZA [relative risk (*RR*) = 7.03, *P* < 0.0001].

**Conclusion:** A meta-analysis of six RCTs suggested that ZA was beneficial in maintaining the periprosthetic BMD in patients with osteoporosis at 6 and 12 months after THA. In addition, the HHS was significantly improved in patients treated with ZA. However, the short length of follow-up of the available studies resulted in the lack of analyses regarding the survival of implants including the rate of aseptic loosing, periprosthetic fracture, and revision. It still needs to be determined in research with longer follow-up period.

**Clinical Trial Registration:** Researchregistry.com, identifier: reviewregistry1087.

## Introduction

Total hip arthroplasty (THA) was an effective treatment for the end-stage hip disease, but periprosthetic bone mineral density (BMD) loss was common following the operation (1) that adversely affect the survival of the implants (2–4). Though the mechanism of periprosthetic BMD loss was not clearly understood, an increase in osteoclast activity and wear particles were thought as two major contributors (4, 5).

Patients undergoing the THA often suffer from osteoporosis (6–8), which has a great influence on the periprosthetic BMD changes and stem fixation after operation. Aro et al. (9) reported that patients with lower bone mass suffered higher stem subsidence during the first 3 months after THA, and osteoporotic hip was a risk factor for delayed translational stability. Therefore, it is necessary to actively treat patients with osteoporosis by anti-osteoporosis medicines for a longer lifespan of stem.

Zoledronic Acid (ZA), as one of the third generation of bisphosphonates, was developed to treat osteoporosis, increase bone density, and reduce the risk of osteoporotic fracture by inhibiting the osteoclastic function and inducing osteoclast apoptosis (10). ZA has been approved by the U.S. Food and Drug Administration for the treatment of patients with osteoporosis (11). In addition, based on the effect of inhibiting bone resorption, it has been proved useful in reducing the periprosthetic BMD loss after total hip replacement (12).

The aim of this meta-analysis is to evaluate the efficacy and safety of a single dose of 5 mg of ZA in patients with osteoporosis after THA. Compared with the first meta-analysis (12), this study was updated on several key points. First, the data of two included studies in that meta-analysis were exactly same including the mean and SD of BMD changes. Therefore, we highly doubt the authenticity of the data in these two articles and exclude them in the process of new literature screening (13, 14). Second, percentage changes of BMD in seven Gruen zones around the stem at 3, 6, and 12 months compared with baseline BMD were pooled in our study. Third, some important outcome parameters, such as Harris hip score (HHS) and adverse events (AEs) were added in this study.

## Materials and Methods

This study has been reported in line with Preferred Reporting Items for Systematic Reviews and Meta-Analyses (PRISMA) (15). This study was based on the previous studies and therefore, no ethical approval and patient consent were required.

### Search Strategy

**Figure 1:**
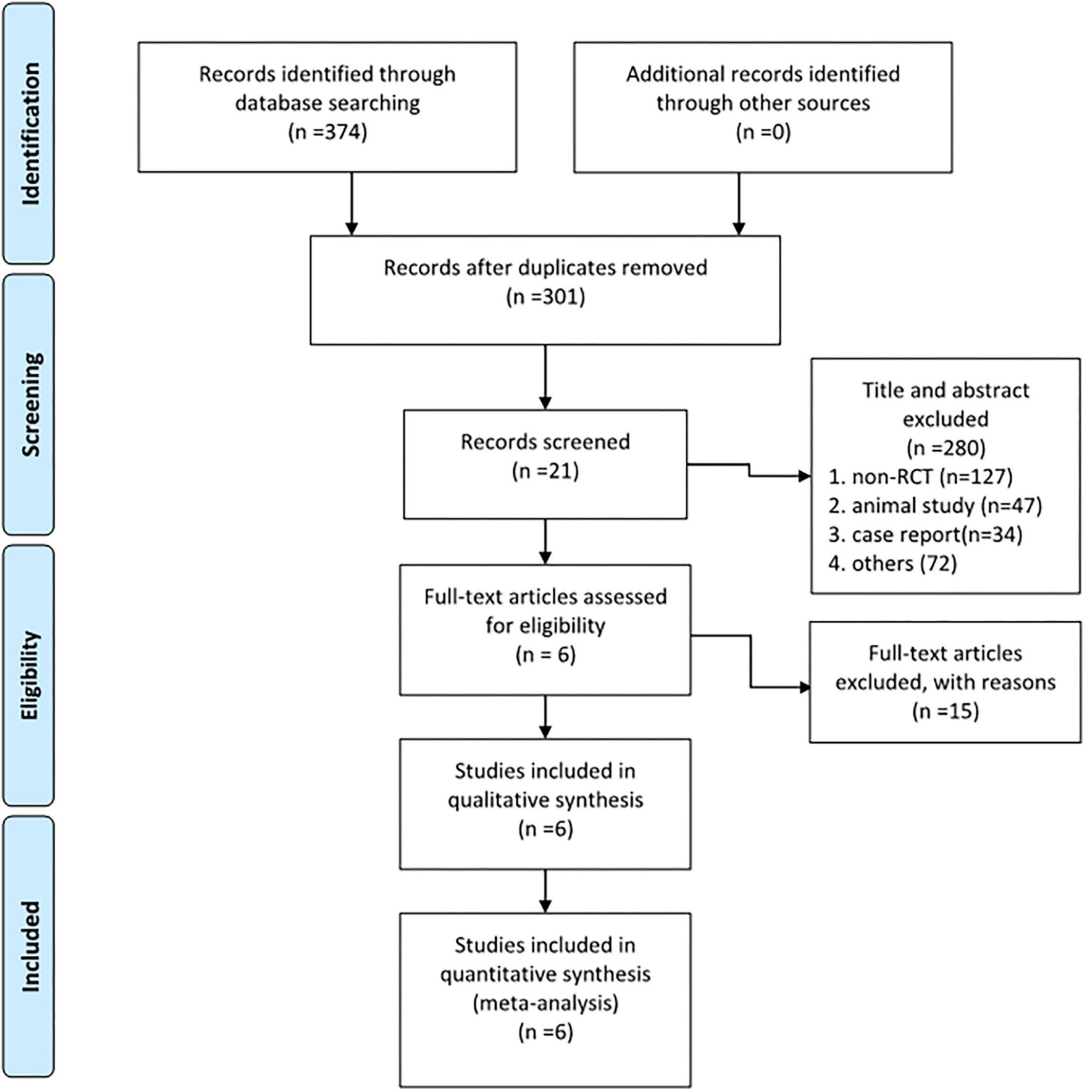
The Preferred Reporting Items for Systematic Reviews and Meta-Analyses (PRISMA) 2009 flow diagram shows 6 RCTs that were included and analyzed.

Databases, such as MEDLINE (1950 to date), PubMed (1966 to date), EMBASE (1974 to date), the Cochrane Central Register of Controlled Trials, the Wan-fang database (1982 to date), and the Web of Science were systematically searched for studies on ZA in total THA in August, 2021. “Hip, hip replacement, hip arthroplasty, total hip replacement, THR, total hip arthroplasty, THA,” and “bisphosphonates, zoledronic acid, zoledronate” were used as keywords in connection with AND or OR. Meta-analyses were identified and screened out of the search results by the reviewer ZY. Then, the references of these meta-analyses were screened to find additional relevant studies. Another reviewer (SB) tried to contact expert informants by email to search for the unpublished studies. Finally, two reviewers (LY and XJW) independently assessed the studies, and any discrepancies were resolved by a discussion. The process of literature screening is shown in Figure 1.

### Inclusion and Exclusion Criteria

Studies were included according to the PICOS criteria: (1) population: patients suffering from osteoporosis undergoing THA who were demographically alike; (2) intervention and control: a single intravenous infusion of ZA or saline solution; (3) outcomes: periprosthetic BMD changes, changes of bone turnover markers, HHS, and AEs; and (4) study design: randomized controlled trial (RCT).

Study was excluded if: (1) non-RCT, (2) cemented stem was used, (3) relevant outcomes were missing, and (4) patients used drugs for osteoporosis or corticosteroids, hepatic or renal disease, skeletal disorder, such as Paget's disease, malignancy within the past 5 years.

### Quality Assessment

The Cochrane Risk of Bias Tool (RoB 2.0) was adopted to assess the methodological quality of the RCTs (16). The tool considers five bias domains, randomized process, deviations from intended interventions, missing outcome data, measurement of the outcome, and selection of the reported result. Based on the Cochrane Handbook, two reviewers (LMY and WLM) independently evaluated the quality of the included RCTs. The disagreement between the ratings of two reviewers was discussed with the third reviewer (ZY).

### Data Extraction

For each eligible study, one of the reviewers (LY) extracted relevant data and another (XJW) checked the accuracy. Authors, year of publication, study design, demographic data [age, sex, and body mass index (BMI)], usage and dose of the ZA, and length of follow-up were extracted using a standard form. Outcomes, such as the periprosthetic BMD changes, biochemical markers of bone turnover, HHS, and AEs were recorded. Percentage changes of periprosthetic BMD in seven Gruen zones at 3, 6, and 12 months after operation compared with baseline BMD was the primary outcome (positive value means the increased BMD and negative value means the decreased BMD). Biochemical markers of bone turnover recorded, such as bone formation markers and bone resorption markers. Only one article was written in Slovak, and it was translated by a medical translator. If the mean BMS was not reported in the text or a table in the article, it was extrapolated from accompanying graphs.

### Evidence Assessment With the Grading of Recommendations, Assessment, Development, and Evaluation (GRADE) Approach

The evidence assessment was determined using the guidelines of the grading of recommendations, assessment, development, and evaluation (GRADE) working group (17). The GRADE system uses a sequential assessment of the evidence quality and the evidence grades are divided into the following levels: (1) high, which indicates that further research is unlikely to alter confidence in the effect estimate; (2) moderate, which indicates that further research is likely to significantly alter confidence in the effect estimate and may change the estimate; (3) low, which indicates that further research is likely to significantly alter confidence in the effect estimate and to change the estimate; and (4) very low, which indicates that any effect estimate is uncertain. Uniformity of the estimated effects across studies and the extent to which the patients, interventions, and outcome measures are similar to those of interest may reduce or increase the evidence grade. As recommended by the GRADE working group, the lowest evidence quality for any of the outcomes was used to rate the overall evidence quality. The evidence quality was graded using GRADEpro online software (https://gradepro.org/).

### Statistical Analysis

The Review Manager 5.3.5 software (The Cochrane Collaboration, Oxford, UK) was used to perform the meta-analysis. Mean difference (MD) was used to weigh the effect size for continuous outcomes, and relative risks (*RR*) were used for dichotomous outcomes. The *I*^2^ statistic was used to test for heterogeneity across the included studies (16). A *p* ≤ 0.1 or an *I*^2^ > 50% was regarded as proof of heterogeneity. A random-effects model was used to alleviate the effect caused by high heterogeneity, and a fixed effects model was used when statistical evidence showed low heterogeneity.

## Results

### Search Results

As shown in Figure 1, among 374 articles that were obtained from the databases *via* the search strategy, 301 articles were screened after removing duplicates. In total, 280 articles were removed after reading the title and abstract based on the inclusion and exclusion criteria. Then, 15 studies were excluded after reading the full text for the irrelevant content or low quality. Finally, 6 RCTs (18–23) were included in this study.

### Study Characteristics and Quality Assessment

Finally, six studies involving 155 patients in ZA group and 152 patients in the control group were included. The mean age was 67.3 and 66.8 years in ZA group and control group, respectively. The mean BMI of patients included in two groups was 26.7 and 26.2 g/cm^2^, respectively. An intravenous infusion of 5 or 4 mg ZA in 100 ml saline solution or the same volume of saline was infused. In addition, other anti-osteoporosis medicines, such as the calcium and vitamin D were supplied daily. The administration time of ZA was mostly within 1 week after operation in included studies, and one was in the second postoperative week and another one was at 2 days before the operation. The length of follow-up ranged 1–4 years (Table 1). Among RCTs, no study of high risk of bias in at least one domain was found (Table 2).

**Table 1:** Characteristics of zoledronic acid (ZA) vs. placebo.

**Studies**	**Study Design**	**Cases (ZA/C)**	**Age (ZA/C)**	**BMI (ZA/C)**	**Female (ZA/C)**	**Time of infusion**	**Zoledronic acid**	**Control**	**Basic anti-osteoporosis drugs**	**Follow-up**
Aro et al. (18)	RCT	24/23	65.3/71	28.4/29.8	-	Three days after operation	A single IV infusion (5 mg)	Saline solution	Calcium and vitamin D	4 years
Friedl et al. (19)	RCT	25/24	63.9/57.8	28.5/28.4	17/10	One day after operation	a single IV infusion (4 mg)	Saline solution	calcium (1000 mg) and vitamin D (400 IU) daily	2.8 years
Huang et al. (20)	RCT	27/27	60.1/59.4	26/25	15/14	One day after operation	A single IV infusion (5 mg)	Saline solution	calcium (600 mg) and vitamin D (800 IU) daily	2 years
Lacko et al. (21)	RCT	15/15	66.8/64.9	27.5/23.4	10/9	The second post-operative week	A single IV infusion (5 mg)	Saline solution	calcium (1000 mg) and vitamin D (880 IU) daily	1 years
Zhou et al. (22)	RCT	16/16	73.3/74.4	23.9/24.4	-	Five–seven days after operation	A single IV infusion (5 mg)	Saline solution	calcium (1200 mg) and calcitriol (0.50 μg) daily	1 years
Zhu et al. (23)	RCT	48/47	74.6/73.1	26/26.4	37/35	Two days before operation	A single IV infusion (5 mg)	Saline solution	calcium (1000 mg) and calcitriol (0.50 μg) daily	1 years

*RCT, randomized controlled trial; BMI, body mass index; IV, intravenous; ZA, zoledronic acid; C, control; Included studies with the symbol – indicating areas that were not reported*.

**Table 2:** Risk of bias assessment for RCTs according to risk of bias (RoB) 2.0.

**Studies**	**Bias domains**					**Overall judgment**
	**Randomized process**	**Deviations from intended interventions**	**Missing outcome data**	**Measurement of the outcome**	**Selection of the reported result**	
Aro et al. (18)	Low risk	Low risk	Low risk	Low risk	Low risk	Low risk
Friedl et al. (19)	Low risk	Low risk	Low risk	Low risk	Low risk	Low risk
Huang et al. (20)	Low risk	Moderate risk	Low risk	Low risk	Low risk	Some concerns
Lacko et al. (21)	Low risk	Low risk	Moderate risk	Low risk	Low risk	Some concerns
Zhou et al. (22)	Low risk	Low risk	Low risk	Low risk	Low risk	Low risk
Zhu et al. (23)	Low risk	Low risk	Low risk	Low risk	Low risk	Low risk

### Periprosthetic BMD Changes

**Figure 2:**
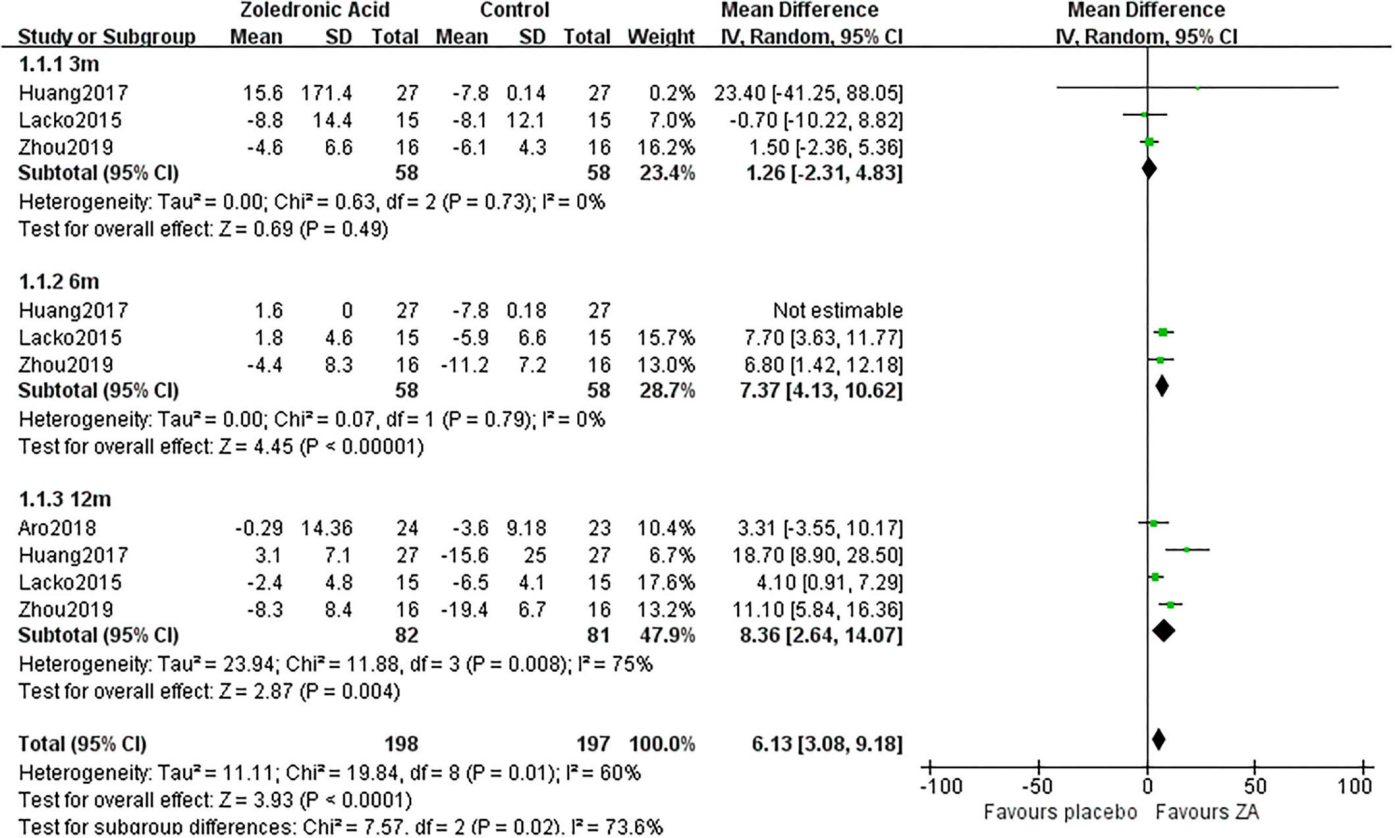
The forest plot shows periprosthetic bone mineral density (BMD) changes between zoledronic acid (ZA) and control group at 3, 6, and 12 months after total hip arthroplasty (THA) in Gruen zone 1.

**Figure 3:**
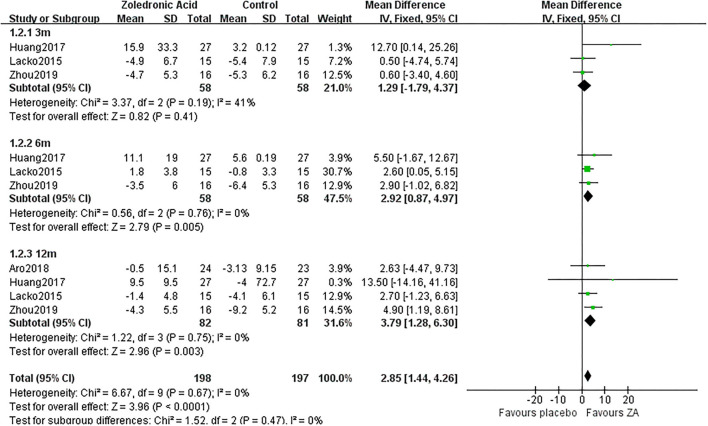
The forest plot shows periprosthetic BMD changes between ZA and control group at 3, 6, and 12 months after THA in Gruen zone 2.

**Figure 4:**
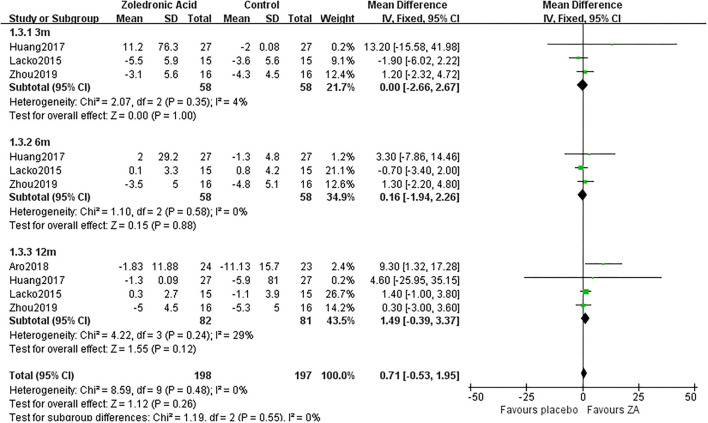
The forest plot shows periprosthetic BMD changes between ZA and control group at 3, 6, and 12 months after THA in Gruen zone 3.

**Figure 5:**
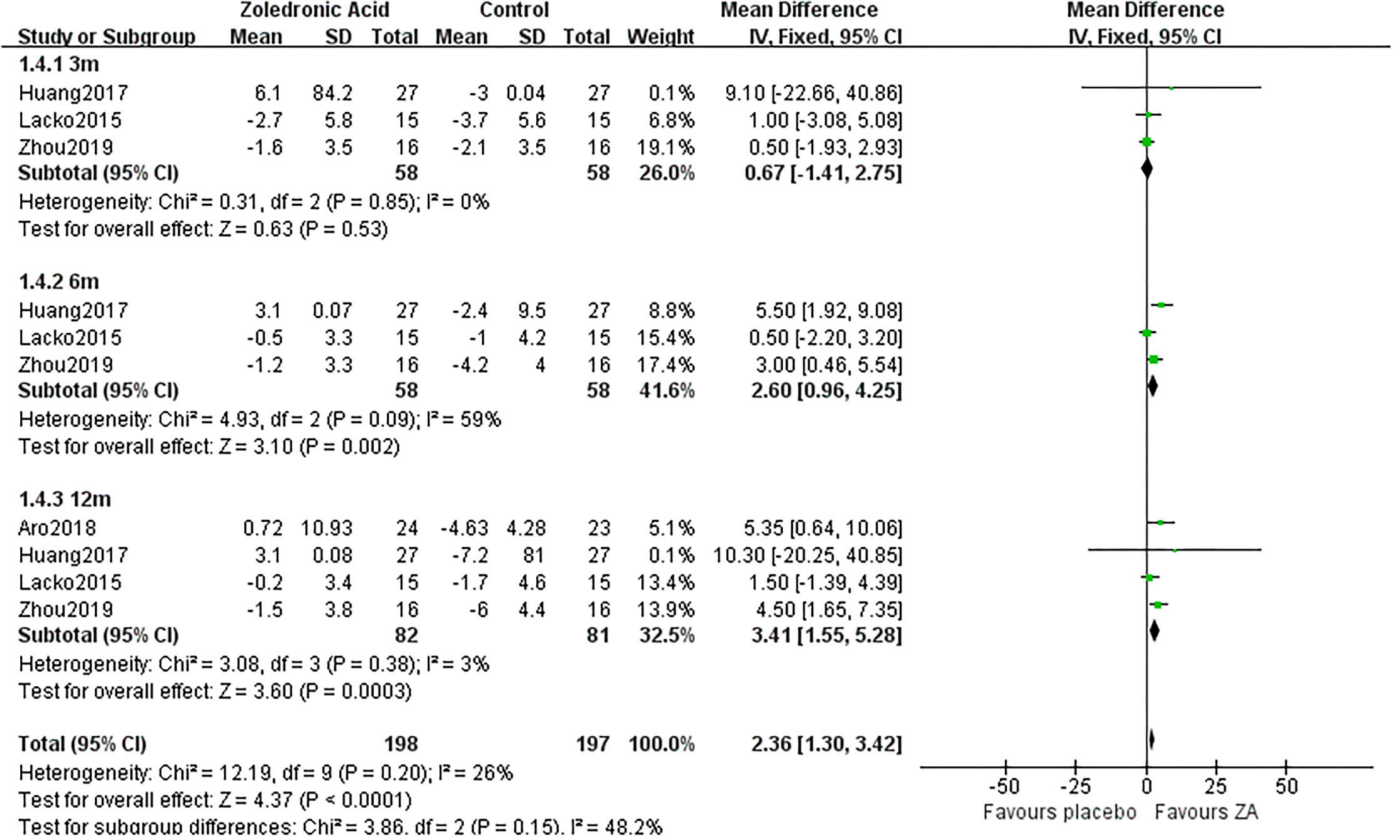
The forest plot shows periprosthetic BMD changes between ZA and control group at 3, 6, and 12 months after THA in Gruen zone 4.

**Figure 6:**
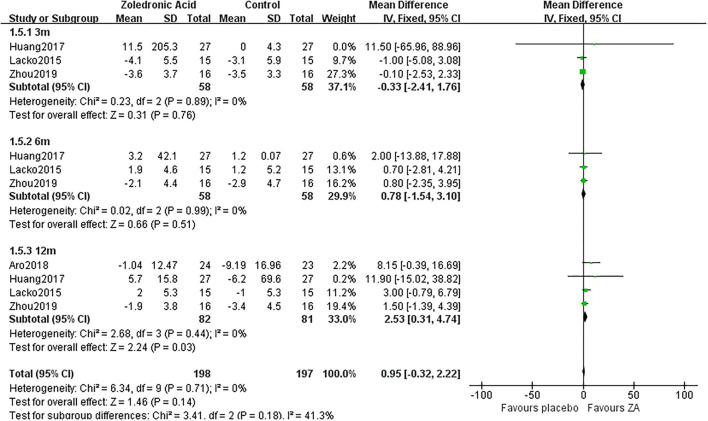
The forest plot shows periprosthetic BMD changes between ZA and control group at 3, 6, and 12 months after THA in Gruen zone 5.

**Figure 7:**
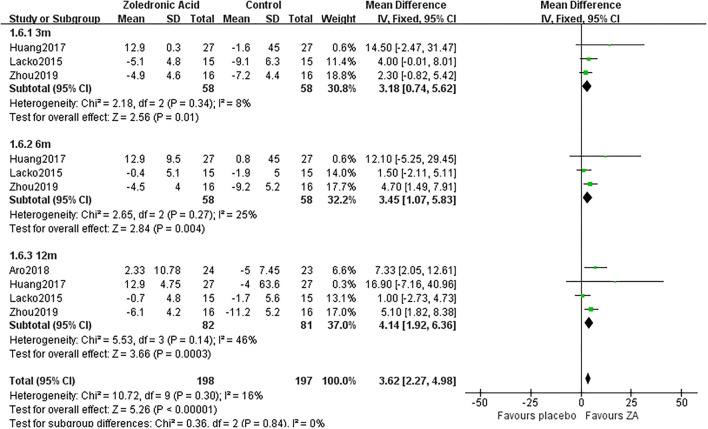
The forest plot shows periprosthetic BMD changes between ZA and control group at 3, 6, and 12 months after THA in Gruen zone 6.

**Figure 8:**
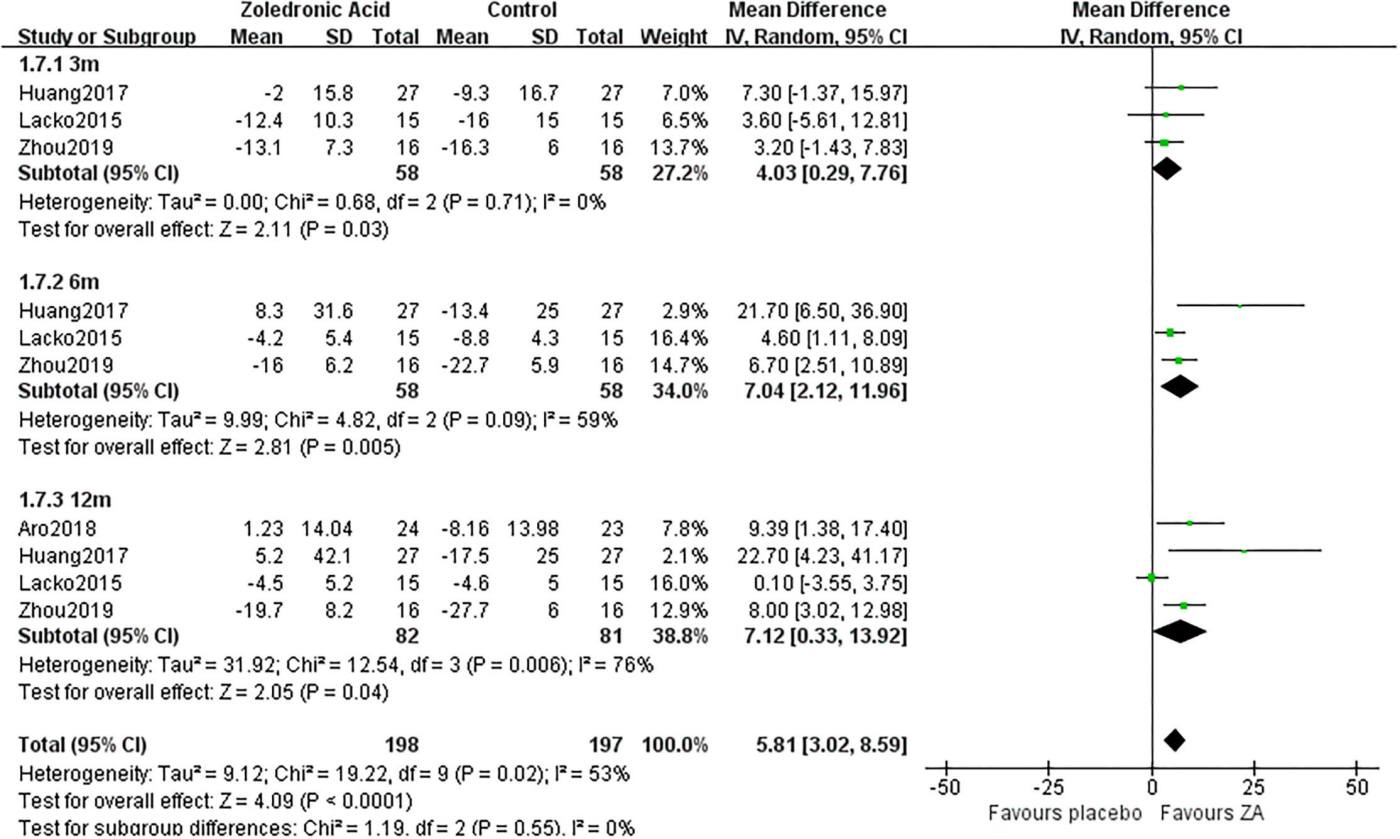
The forest plot shows periprosthetic BMD changes between ZA and control group at 3, 6, and 12 months after THA in Gruen zone 7.

The periprosthetic BMD changes were assessed by the dual-energy X-ray absorptiometry (DEXA) in included RCTs. Three RCTs (20–22) involving 58 patients in each group compared the periprosthetic BMD changes at 3 and 6 months after THA, and four RCTs (17–21) involving 82 patients in ZA group and 81 patients in control group reported the periprosthetic BMD changes at 12 months after THA. The forest plot analyzed the periprosthetic BMD changes at 3, 6, and 12 months after THA in Gruen zones 1–7 were shown in Figures 2–8. Overall, the periprosthetic BMD loss was significantly relieved by the usage of ZA at 6 and 12 months after THA, especially in the Gruen zone 1 and 7 (Table 3, Supplementary Figure 1).

**Table 3:** Results of meta-analysis about the periprosthetic bone mineral density (BMD) changes between ZA and control group.

**Gruen zones**	**Time points**	**Studies**	**Patients (ZA/C)**	**Heterogeneity (I^**2**^, %)**	**Model**	**MD (95% CI)**	***P*-value**
Gruen 1	3 m	3	58/58	0%	Fixed	1.26 [−2.31, 4.83]	0.49
	6 m	3	58/58	0%	Fixed	7.37 [4.13, 10.62]	<0.00001
	12 m	4	82/81	75%	Random	8.36 [2.64, 14.07]	0.004
	3 m	3	58/58	41%	Fixed	1.29 [−1.79, 4.37]	0.41
Gruen 2	6 m	3	58/58	0%	Fixed	2.92 [0.87, 4.97]	0.005
	12 m	4	82/81	0%	Fixed	3.79 [1.28, 6.30]	0.003
	3 m	3	58/58	4%	Fixed	0.00 [−2.66, 2.67]	1
Gruen 3	6 m	3	58/58	0%	Fixed	0.16 [−1.94, 2.26]	0.88
	12 m	4	82/81	29%	Fixed	1.49 [-0.39, 3.37]	0.12
	3 m	3	58/58	0%	Fixed	0.67 [−1.41, 2.75]	0.53
Gruen 4	6 m	3	58/58	59%	Random	2.81 [0.17, 5.44]	0.04
	12 m	4	82/81	3%	Fixed	3.41 [1.55, 5.28]	0.0003
	3 m	3	58/58	0%	Fixed	0.78 [−1.54, 3.10]	0.76
Gruen 5	6 m	3	58/58	0%	Fixed	2.53 [0.31, 4.74]	0.51
	12 m	4	82/81	0%	Fixed	0.95 [−0.32, 2.22]	0.03
	3 m	3	58/58	8%	Fixed	3.18 [0.74, 5.62]	0.01
Gruen 6	6 m	3	58/58	25%	Fixed	3.45 [1.07, 5.83]	0.004
	12 m	4	82/81	46%	Fixed	4.14 [1.92, 6.36]	0.0003
	3 m	3	58/58	0%	Fixed	4.03 [0.29, 7.76]	0.03
Gruen 7	6 m	3	58/58	59%	Random	7.04 [2.12, 11.96]	0.005
	12 m	4	82/81	76%	Random	7.12 [0.33, 13.92]	0.04

*The larger the mean difference (MD) was, the more bone preserved by the ZA*.

*ZA, zoledronic acid; C, control group; MD, mean difference*.

### Periprosthetic BMD Changes in Gruen Zone 1

Zoledronic acid significantly preserved more BMD in Gruen zone 1 compared with the control group at 6 months (MD = 7.37, 95% *CI*: 4.13–10.62, *P* < 0.00001) and 12 months (MD = 8.36, 95% *CI*: 2.64–14.07, *P* = 0.04) after THA. While there was no significant difference between two groups in Gruen zone 1 at 3 months after THA (Figure 2).

### Periprosthetic BMD Changes in Gruen Zone 2

Zoledronic acid significantly preserved more BMD in Gruen zone 2 compared with the control group at 6 months (MD = 2.92, 95% *CI*: 0.87–4.97, *P* = 0.005) and 12 months (MD = 3.79, 95% *CI*: 1.28–6.3, *P* = 0.003) after THA. While there was no significant difference between two groups in Gruen zone 2 at 3 months after THA (Figure 3).

### Periprosthetic BMD Changes in Gruen Zone 3

There was no significant difference between two groups in Gruen zone 3 at 3, 6, and 12 months after THA (Figure 4).

### Periprosthetic BMD Changes in Gruen Zone 4

Zoledronic acid significantly preserved more BMD in Gruen zone 4 compared with the control group at 6 months (MD = 2.6, 95% *CI*: 0.96–4.25, *P* = 0.002) and 12 months (MD = 3.41, 95% *CI*: 1.55–5.28, *P* = 0.0003) after THA. While there was no significant difference between two groups in Gruen zone 4 at 3 months after THA (Figure 5).

### Periprosthetic BMD Changes in Gruen Zone 5

Zoledronic acid significantly preserved more BMD in Gruen zone 5 compared with the control group at 12 months (MD = 2.53, 95% *CI*: 0.31–4.74, *P* = 0.03) after THA. While there was no significant difference between two groups in Gruen zone 5 at 3 and 6 months after THA (Figure 6).

### Periprosthetic BMD Changes in Gruen Zone 6

Zoledronic acid significantly preserved more BMD in Gruen zone 6 compared with the control group at 3 months (MD = 3.18, 95% *CI*: 0.74–5.62, *P* = 0.01), 6 months (MD = 3.45, 95% *CI*: 1.07–5.83, *P* = 0.004), and 12 months (MD = 4.14, 95% *CI*: 1.92–6.36, *P* = 0.0003) after THA (Figure 7).

### Periprosthetic BMD Changes in Gruen Zone 7

Zoledronic acid significantly preserved more BMD in Gruen zone 6 compared with the control group at 3 months (MD = 4.03, 95% *CI*: 0.29–7.76, *P* = 0.03), 6 months (MD = 7.04, 95% *CI*: 2.12–11.96, *P* = 0.005), and 12 months (MD= 7.12, 95% *CI*: 0.33–13.92, *P* = 0.04) after THA (Figure 8).

### Biochemical Markers of Bone Turnover

**Figure 9:**
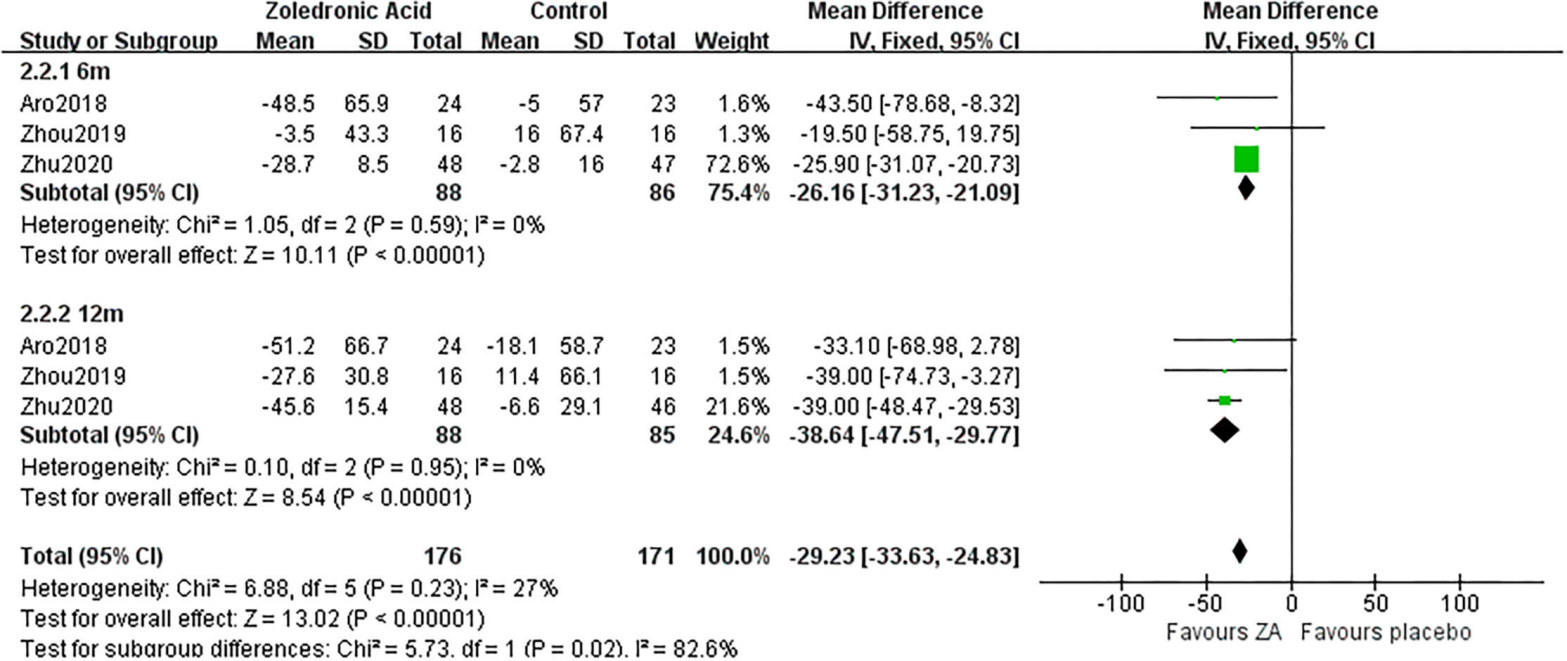
The forest plot shows the changes of procollagen type I N-terminal propeptide (PINP) between ZA and control group at 6 and 12 months after THA.

Three RCTs (18, 22, 23) involving 88 patients in ZA group and 86 patients in control group reported the changes of procollagen type I N-terminal propeptide (PINP) at 6 and 12 months after THA. The pooled result demonstrated PINP was significantly decreased in ZA group compared with control group at 6 and 12 months after THA (MD = −26.16, 95% *CI*: −31.23 to −21.09, *P* < 0.00001; MD = −38.64, 95% *CI*: −47.51 to −29.77, *P* < 0.00001, Figure 9). Additionally, only three of six RCTs (18, 20, 23) mentioned that blood vitamin D and calcium levels were normal before administrating ZA.

### Harris hip Score

**Figure 10:**
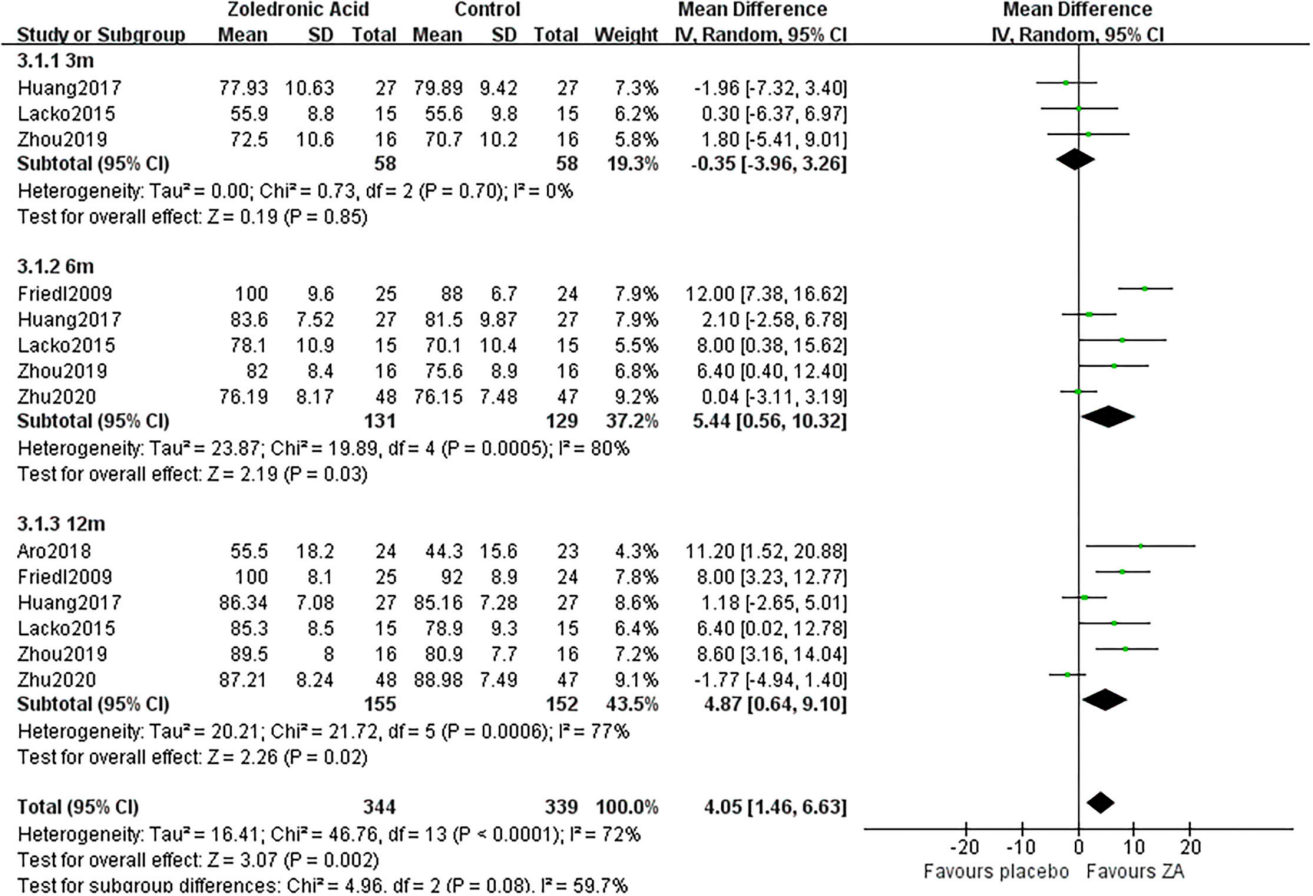
The forest plot shows the Harris hip score (HSS) between ZA and control group at 3, 6, and 12 months after THA.

Three RCTs (20–22) involving 58 patients in each group recorded the HHS at 3 months after THA. The pooled results showed that the HHS was not significantly different between two groups at 3 months after THA (MD = −0.35, 95% *CI*: −3.96 to 3.26, *P* = 0.85, Figure 10).

Five RCTs (19–23) involving 131 patients in ZA group and 129 patients in the control group recorded the HHS at 6 months after THA. The pooled results showed that the HHS was significantly increased in the ZA group compared with the control group at 6 months after THA (MD = 5.44, 95% *CI*: 0.56–10.32, *P* = 0.03, Figure 10).

Six RCTs (18–23) involving 155 patients in the ZA group and 152 patients in the control group recorded the HHS at 12 months after THA. The pooled results showed that the HHS was significantly increased in the ZA group compared with the control group at 12 months after THA (MD = 4.87, 95% *CI*: 0.64–9.1, *P* = 0.02, Figure 10).

### Adverse Events

**Figure 11:**
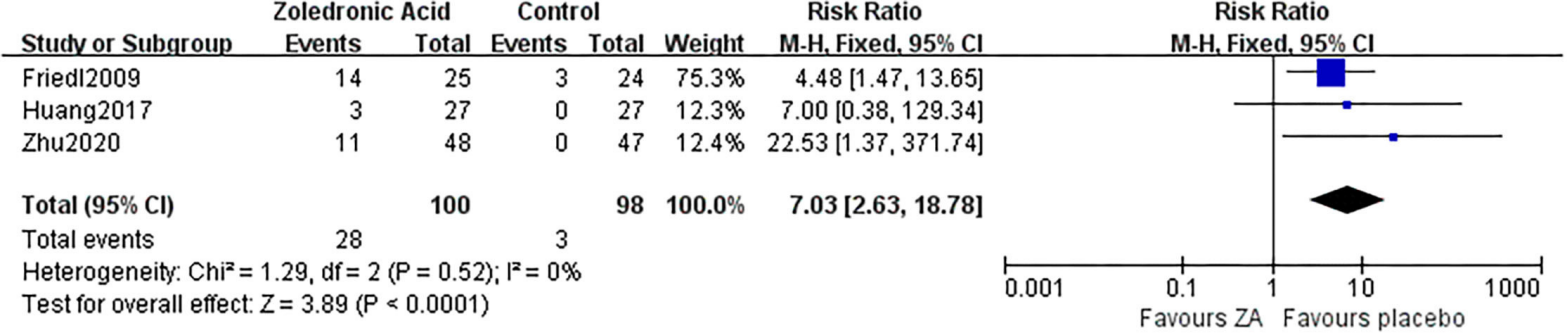
The forest plot shows the rate of influenza-like symptom between ZA and control group after THA.

No serious or fatal AE was reported in the 6 RCTs. While influenza-like symptom was reported in three RCTs involving 100 patients in the ZA group and 98 patients in the control group. There were 28 patients (28%) in the ZA group and three patients (3.1%) in the control group suffered influenza-like symptom, which was significantly different (*RR* = 7.03, 95% *CI*: 2.63–18.78, *p* < 0.0001, Figure 11).

### Quality of the Evidence in the GRADE System

As shown in Supplementary Table 1, a total of 10 outcomes in this meta-analysis were evaluated using the GRADE system. The quality of evidence in the half of 10 outcomes was high: periprosthetic BMD changes in Gruen zone 1 at 3 and 6 months after THA, zone 7 and HHS at 3 months after THA and influenza-like symptoms. The remaining five outcomes had moderate quality of evidence. Therefore, we believed that the overall evidence quality of our meta-analysis was very moderate.

## Discussion

Periprosthetic BMD loss was common and related with aseptic loosening, periprosthetic fracture, and revision after THA (24). Several studies have reported that bisphosphonates were effective in reducing the periprosthetic BMD loss and increased the survival time of implants (25–27). In this study, the efficacy of a single intravenous infusion of ZA (5 mg) on patients with osteoporosis undergoing THA was evaluated. The pooled results based on the currently available literature provided evidence that ZA not only significantly preserved more bone mass at 6 and 12 months after THA, but also improved HSS. In addition, one (19) of three RCTs evaluated the effect of ZA on stem migration concluded that ZA effectively minimized the migration of the cups stem subsidence.

As the third-generation bisphosphonates, annual intravenous infusion of zoledronate has been proven useful in treating osteoporosis *via* suppressing osteoclasts activities (28–30). Oral bisphosphonates daily or weekly have been reported to have long-term effects in the preservation of periprosthetic BMD after joint arthroplasty (31). While the efficacy was depending on the daily administration, the zoledronic acid was just needed to be infused once a year. Nevertheless, it needed to be noted that ZA may not be effective in all patients with osteoporosis. Moller et al. (32) have reported that patients exhibit a variable sensitivity to ZA. Therefore, the alternative bisphosphonates and sequential therapy should be considered when necessary (33).

The improved HHS in our study illustrated the positive effect of ZA on the functional recovery in patients with osteoporosis after THA. On the one hand, it inhibited the periprosthetic bone loss resulting from the stress shielding, enhanced the fixation, and stability of implants. On the other hand, it improved the BMD of the whole body, that was helpful for improving the life quality in patients with osteoporosis.

The safety of the ZA was equally important. Osteonecrosis of the jaw was considered as the worst complication of bisphosphonate therapy (34, 35), and though its incidence was low, the supervision of a dentist might be necessary. In addition, atypical femoral fractures were reported in patients who had long-term treatment with bisphosphonates (36). However, contrary to these reports, only influenza-like symptom was found in our study and could be alleviated by symptomatic treatment. Therefore, whether the usage of ZA brings other serious complications in patients with osteoporosis still needs further research.

There are several limitations to this meta-analysis. First, the subgroup analysis regarding administration time of ZA was not made for only one study administered before the operation, the remaining five studies were all infused within 2 weeks after operation. It has been hypothesized that systemically administrated bisphosphonates accumulate locally in freshly exposed bone mineral after drilling and reaming during the implantation of a hip prosthesis (37). Therefore, it seemed that it was better to infuse during the days of operation. Second, the length of follow-up was too short to evaluate the outcomes related to implants survival. However, the main objective of this study was to investigate the periprosthetic BMD changes in patients treated with ZA at 6 and 12 months after THA. Since the periprosthetic BMD loss was most evident in the first postoperative year and the changes were minimal thereafter (1, 38). It was proposed that the changes in the first year were more clinically relevant, as the initial periprosthetic bone remodeling process was mainly completed in the first 12 postoperative months (39, 40). Thus, 1–2 years is generally considered as an adequate follow-up for the evaluation of early-stage periprosthetic bone remodeling (13). As for the effect of ZA on the rate of aseptic loosing, periprosthetic fracture and implant survival was expected to be determined in future studies with prolonged follow-up period.

## Conclusion

A meta-analysis of six randomized controlled trials suggested that ZA was beneficial in maintaining periprosthetic BMD in patients with osteoporosis at 6 and 12 months after THA. The HHS was significantly improved in patients treated with ZA along with the more BMD preserved. However, the short length of follow-up of the available studies resulted in the lack of analyses regarding the survival of implants including the rate of aseptic loosing, periprosthetic fracture, and revision. It still needs to be determined in research with longer follow-up period.

## Data Availability Statement

The original contributions presented in the study are included in the article/Supplementary Material, further inquiries can be directed to the corresponding author.

## Author Contributions

BS: designed the study. YL and J-WX: gathered the data. M-YL and L-MW: analyzed the data. YL: wrote the initial drafts. YZ and BS: ensure the accuracy of the data and analysis. All authors contributed to the article and approved the submitted version.

## Funding

This study was supported through grants from the National Natural Science Foundation of China (81974347) and the National Clinical Research Center for Geriatrics, West China Hospital Sichuan University (No. Z20191008).

## Conflict of Interest

The authors declare that the research was conducted in the absence of any commercial or financial relationships that could be construed as a potential conflict of interest.

## Publisher's Note

All claims expressed in this article are solely those of the authors and do not necessarily represent those of their affiliated organizations, or those of the publisher, the editors and the reviewers. Any product that may be evaluated in this article, or claim that may be made by its manufacturer, is not guaranteed or endorsed by the publisher.
