# Supplementary material for: Zoledronic Acid for Periprosthetic Bone Mineral Density Changes in Patients With Osteoporosis After Hip Arthroplasty—An Updated Meta-Analysis of Six Randomized Controlled Trials
Source: Front Med (Lausanne). 2021 Dec 23;8:801282. doi: 10.3389/fmed.2021.801282 (PMC8733298; doi:10.3389/fmed.2021.801282)
Supplement: Supplementary file 1 [file Data_Sheet_1.PDF]

Supplementary material

S-Fig. 1 The effect of the zoledronic acid on the bone mineral density changes in Gruen zones according to the meta-analysis from 6 RCTs.

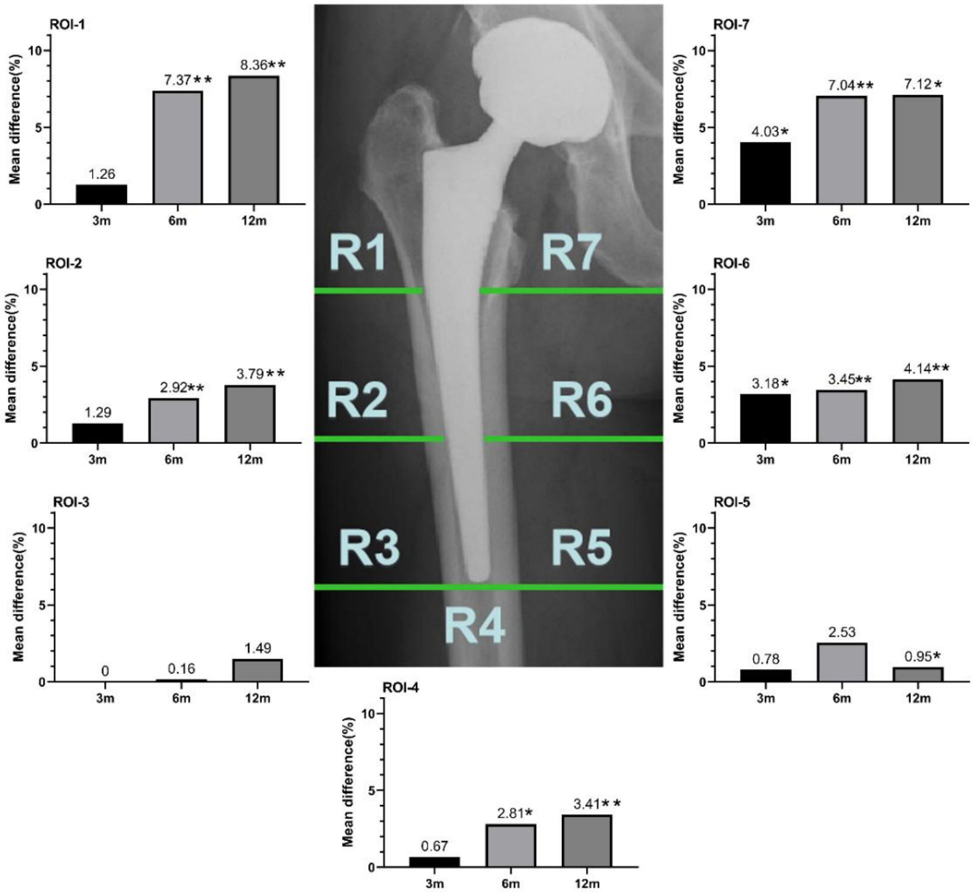

Mean difference was the D-value of the periprosthetic bone mineral density changes between the zoledronic acid and control group. *p < 0.05, **p < 0.01.

Table 4 The GRADE evidence quality for significant outcomes

Certainty assessment							№ of patients		Effect		Certainty	Importance
**№ of studies**	**Study design**	**Risk of bias**	**Inconsistency**	**Indirectness**	**Imprecision**	**Other considerations**	**ZA**	**Control**	**Relative (95% CI)**	**Absolute (95% CI)**		
Gruen 1 - 3m												
3	randomised trials	not serious	not serious	not serious	not serious	none	58	58	-	MD 1.26 higher (2.31 lower to 4.83 higher)	⊕⊕⊕⊕ HIGH	
Gruen 1 - 6m												
3	randomised trials	not serious	not serious	not serious	not serious	none	58	58	-	MD 7.37 higher (4.13 higher to 10.62 higher)	⊕⊕⊕⊕ HIGH	
Gruen 1 - 12m												
4	randomised trials	not serious	serious [a]	not serious	not serious	none	82	81	-	MD 8.36 higher (2.64 higher to 14.07 higher)	⊕⊕⊕◯ MODERATE	
Gruen 7 - 3m												
3	randomised trials	not serious	not serious	not serious	not serious	none	58	58	-	MD 4.03 higher (0.29 higher to 7.76 higher)	⊕⊕⊕⊕ HIGH	
Gruen 7 - 6m												
3	randomised	not	serious [a]	not serious	not serious	none	58	58	-	MD 7.04 higher	⊕⊕⊕◯	

	trials	serious								(2.12 higher to 11.96 higher)	MODERATE
Gruen 7 - 12m											
4	randomised trials	not serious	serious [a]	not serious	not serious	none	82	81	-	MD 7.12 higher (0.33 higher to 13.92 higher)	⊕⊕⊕◯ MODERATE
HHS - 3m											
3	randomised trials	not serious	not serious	not serious	not serious	none	58	58	-	MD 0.35 lower (3.96 lower to 3.26 higher)	⊕⊕⊕⊕ HIGH
HHS - 6m											
5	randomised trials	not serious	serious [a]	not serious	not serious	none	131	129	-	MD 5.44 higher (0.56 higher to 10.32 higher)	⊕⊕⊕◯ MODERATE
HHS - 12m											
6	randomised trials	not serious	serious [a]	not serious	not serious	none	155	152	-	MD 4.87 higher (0.64 higher to 9.1 higher)	⊕⊕⊕◯ MODERATE
influenza-like symptoms											
3	randomised trials	not serious	not serious	not serious	not serious	none	28/100 (28.0%)	3/98 (3.1%)	RR 7.03 (2.63 to 18.78)	185 more per 1,000 (from 50 more to 544 more)	⊕⊕⊕⊕ HIGH

ZA: zoledronic acid; **CI:** Confidence interval; **MD:** Mean difference; **RR:** Risk ratio

a. heterogeneity was high
